# Lumican Binds ALK5 to Promote Epithelium Wound Healing

**DOI:** 10.1371/journal.pone.0082730

**Published:** 2013-12-18

**Authors:** Osamu Yamanaka, Yong Yuan, Vivien Jane Coulson-Thomas, Tarsis Ferreira Gesteira, Mindy K. Call, Yujin Zhang, Jianhua Zhang, Shao-Hsuan Chang, Changchun Xie, Chia-Yang Liu, Shizuya Saika, James V. Jester, Winston W-Y Kao

**Affiliations:** 1 Deparment of Ophthalmology, University of Cincinnati, Cincinnati, Ohio, United States of America; 2 Division of Developmental Biology, Cincinnati Children's Hospital Research Foundation, Cincinnati, Ohio, United States of America; 3 Department of Environmental Health, University of Cincinnati, Cincinnati, Ohio, United States of America; 4 Department of Ophthalmology, Wakayama Medical College, 811-1 Kimiidera, Wakayama, Japan; 5 Gavin Herbert Eye Institute, Ophthalmology, University of California Irvine, Irvine, California, United States of America; University of Reading, United Kingdom

## Abstract

Lumican (Lum), a small leucine-rich proteoglycan (SLRP) family member, has multiple matricellular functions both as an extracellular matrix component and as a matrikine regulating cell proliferation, gene expression and wound healing. To date, no cell surface receptor has been identified to mediate the matrikine functions of Lum. This study aimed to identify a perspective receptor that mediates Lum effects on promoting wound healing. Transforming growth factor-β receptor 1 (ALK5) was identified as a potential Lum-interacting protein through *in silico* molecular docking and molecular dynamics. This finding was verified by biochemical pull-down assays. Moreover, the Lum function on wound healing was abrogated by an ALK5-specific chemical inhibitor as well as by ALK5 shRNAi. Finally, we demonstrated that eukaryote-specific post-translational modifications are not required for the wound healing activity of Lum, as recombinant GST-Lum fusion proteins purified from *E. coli* and a chemically synthesized LumC_13_ peptide (the last C-terminal 13 amino acids of Lum) have similar effects on wound healing *in vitro* and *in vivo*.

## Introduction

Lumican (Lum) belongs to the small leucine rich proteoglycan (SLRP) family. Like most small leucine rich proteoglycans, the core protein of Lum has a molecular weight of ∼40 kDa and is composed of four major domains: (1) a signal peptide of 16 residues; (2) a negatively charged N-terminal domain containing sulfated tyrosine and four conserved cysteine residues forming intra-chain disulfide bond(s); (3) a tandem leucine-rich repeat region (LXXLXLXXNXLSXL)_10,_ which mediates binding to other extracellular components, e.g., collagen and (4) a C-terminal domain of 53 amino acids containing two conserved cysteine residues 32 amino acid residues apart. Lum is ubiquitously expressed by most cells of connective tissue, if not all. In most connective tissues, it exists as a non-sulfated glycoprotein, except in the corneal stroma where it is one of the major keratan sulfate proteoglycans besides keratocan (Kera) and mimecan [1], [2]. Healing corneal epithelium also express Lum after epithelial debridement [3].

Many SLRP members, e.g., decorin (Dcn) and biglycan (Bgn), possess multiple matricellular functions serving as components of the extracellular matrix (ECM) as well as matrikines in physiological and pathophysiological conditions [2], [4]–[9]. For example, Dcn regulates collagen fibrillogenesis by bridging type I & II collagen fibrils to other regulatory collagens [10]–[13] and binds extracellular molecules [14], [15] as well as growth factors, e.g., TGFβ, TGFα, VEGF [16]–[18]. Dcn also binds to both EGFR [19]–[21] and insulin-like growth factor receptor regulating tumorigenesis, cell proliferation and apoptosis [22]. Bgn has also been shown to be a matrikine *via* binding growth factors such as TGFα [23] and TGFβ [4], [24], as well as modulating Toll like receptor 4 (TLR4) signaling [25], Chordin/BMP [26], [27] and Rho/Rac1 [8].

Lum also has multiple matricellular functions. As a constituent of the ECM, Lum serves as a regulator of collagen fibrillogenesis for the formation and maintenance of transparent corneas and the integrity of many other connective tissues, e.g., sclera, skin [28]–[31] and as a chemokine gradient maker by sequestering CXCL1 during corneal inflammation [32]–[35]. As a matrikine Lum also promotes corneal epithelial wound healing [3], modulates epithelium-mesenchyme transition during the healing of an injured lens and regulates the gene expression profile of stromal keratocytes [32], [36]–[38]. Chakravarti and her co-workers have suggested that Lum may interact with TLR4 to mediate its functions in inflammation [39]–[41]. However, the cellular and molecular mechanism of Lum in promoting wound healing, gene expression and modulating inflammation *via* TLR4 remains largely unknown. It has been hypothesized that there is a cell surface receptor for Lum which mediates some of its matrikine functions other than mediating the aforementioned innate immunity via TLR4 [2]. Funderburgh and co-workers have suggested a cell surface receptor on macrophages, which specifically binds un-sulfated Lum core protein, but not the KS-Lum (keratan-sulfate-lumican) [42]. Nevertheless, the nature of such receptor(s) is not known.

In this study, we found recombinant Lum purified from *E. coli* promoted the wound healing of scratched human telomerase immortalized corneal epithelial (HTCE) cells accompanied by sustained activation of pERK1/2 and lifted the cell cycle suppression at the wound edge. Molecular dynamics studies revealed that ALK5 binds Lum *via* the interaction of the C-terminal 50 amino acids of Lum (LumC_50_). This finding was verified by pull-down assays. The effect of Lum on HTCE wound healing was abrogated by both an ALK5-specific chemical inhibitor (SB431542) and by ALK5-shRNAi. We also found that peptides from the C-terminal domain of Lum can promote wound healing both *in vitro* and *in vivo*, making them promising therapeutic reagents for epithelium wound healing.

## Results

### Recombinant Lum promoted healing of scratched HTCE cells

**Figure 1:**
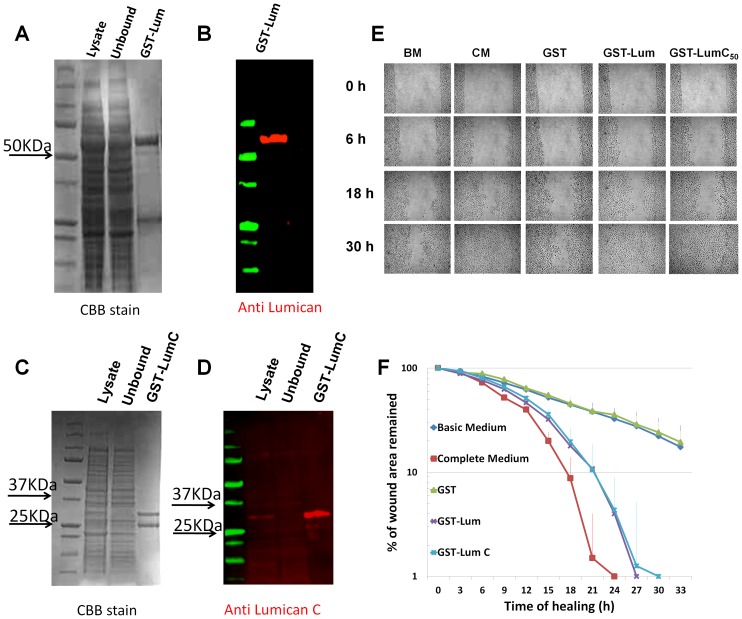
Purification of recombinant Lumican and the healing of scratched HTCE cells. *Purification of GST-Lum and GST-LumC_50_ recombinant proteins* Purification of recombinant GST-Lum and GST-LumC_50_ was monitored by Coomassie Brilliant Blue (CBB) staining and western blot anlaysis. (A) CBB staining revealed two major bands eluted with glutathione. The upper band with a Mr. ∼70 kDa was GST-Lum and the lower band with a Mr. ∼25 kDa was GST. (B) Immunostaining with an anti-LumN oilgopeptide antibody (CDDLKLKSVPMVPPGIK) only labeled the 70 kDa band (GST-Lum fusion protein) while the lower band did not react to the antibody and is likely related to GST. (C) CBB stained two bands at 30 and 25 kDa from *E.coli* transfected with GST-LumC_50_ plasmid. (D) Immunostaining with an anti-LumC peptide antibody (NPLTQSSLPPDMYEC) labeled the 30 kDa GST-LumC_50_ fusion protein. *Effect of recombinant GST-Lum and GST-LumC_50_ on healing of scratched HTCE cells* Confluent HTCE cells were wounded in CM (complete medium), BM (basic medium), BM + GST (glutathione S-transferase recombinant protein), BM+recombinant GST-Lum (0.15 µM) and GSTLumC_50._ The wound gap was determined by time-lapse microscopy. (E) Representative time-lapse images of the healing of scratched HTCE cells; (F) The healing followed biphasic kinetics in cells treated with BM+GST-Lum and CM, whereas those of BM and BM+GST followed monophasic kinetics. R^2^ values were as follows: BM 0.957; CM 0.994; GST 0.985; LumC_50_ 0.985; Lum 0.995. The rate constants are summarized in Table 1.

In order to study the underlying mechanisms of how Lum promotes wound healing, we have to develop an assay in which the signal pathways can be easily manipulated for loss-of-function studies. The assay should be able to recapitulate most of the functions of Lum, such as promoting cell proliferation and migration, observed in *ex vivo* organ culture and *in vivo*. We previously reported that Lum purified from amniotic membrane promotes wound healing following corneal epithelium debridement [38], but it is not clear if glycosylation is required for its activity. We have prepared recombinant GST-Lum fusion proteins (GST-Lum and GST-LumC_50_ containing the C-terminal 50 amino acids of Lum) from *E. coli*, which lacks the eukaryote-specific post-translational modifications, including glycosylation. As shown in Fig. 1A-D, both Coomassie Brilliant Blue staining and Lum-specific Western blotting revealed that purified GST-Lum presented as a single band at the predicted molecular weight of 73 kDa and 30 kDa for GST-Lum and GST-LumC_50_, respectively. A band with a molecular weight of 25 kDa representing GST was also co-purified by the glutathione column. Next, we tested if recombinant Lum purified from *E. coli* could still promote wound healing. First, different doses of GST-Lum were used in a wound healing assay of scratched HTCE cells. It was found that 10 µg/ml (150 nM) GST-Lum can significantly promote healing of scratched HTCE cells, while 1 µg/ml had little effect on wound healing and 100 µg/ml had adverse effects on HTCE cells. A similar dosage was reported in wound healing activity of Lum purified from amniotic membrane [38]. Thereafter, 150 nM of GST-Lum and equimolar concentration of Lum derivatives were used in subsequent experiments. Fig. 1E represents time-lapse images of the healing HTCE cells under different conditions. These images were used to draw the healing plot (Fig. 1F), which demonstrated that the recombinant GST-Lum fusion protein promoted wound healing with biphasic kinetics similar to that seen in cells treated with complete medium (CM) containing pituitary gland extracts and TGFα, whereas those treated with basic medium (BM) only supplemented with glutamine and GST followed monophasic kinetics (Table 1).

**Table 1:** Effect of Lum, LumC_50_ and LumC-peptides on wound healing of HTCE cells.

Culture condition	Rate constant 1 Δ%/h	Rate constant 2 Δ%/h
Basic medium (9)	3.37±0.71	N.A.
Complete medium (8)	6.12±0.85(P<0.01)^a^	16.86±1.71
GST (9)	3.39±0.68(P = 0.95)^a^	N.A.
GST-Lum (7)	5.37±1.17(P<0.01)^a^	14.83±1.61
GST-LumC_50_ (8)	5.35±1.21(P<0.01)^a^ (P = 0.97)^b^	14.04±1.34(P = 0.98)^c^
LumC_33_Δ_C20_ (3)	3.45±0.33(P = 0.87)^a^ (P<0.05)^b^	N.A.
LumC_18_Δ_C5_ (3)	3.62±0.42(P = 0.59)^a^ (P<0.05)^b^	N.A.
LumC_13_ (5)	4.62±0.68(P<0.01)^a^ (P = 0.23)^b^	13.5±2.13(P = 0.28)^c^
LumC_13C-A_ (5)	5.1±0.85 (P<0.01)^a^ (P = 0.67)^b^	13.95±2.38(P = 0.59)^c^

^a^ compared to BM; ^b,c^ compared to GST-Lum. Numbers in parenthesis represent the number of experiments performed. P value <0.05 is considered significant. NA – not applicable. Statistical analysis using one way ANOVA:

Lum and Kera are sister core proteins of the SLRP family found in the corneal stroma. Our previous studies of *Lum* ^−/−^ and *Kera* ^−/−^ null mice demonstrated that Lum modulates the expression of Kera, but not vice versa [32]. Analysis of amino acid sequence alignment indicates that the major sequence diversity is located within the C-terminal domains of the two core proteins, implicating that the LumC terminal domain may account for the wound healing effects of Lum. To examine this possibility, a GST-LumC_50_ fusion protein containing the last 50 amino acids of Lum was tested for its effects on the healing of scratched HTCE cells. As shown in Fig. 1F, GST-LumC_50_ does indeed promote wound healing similar to that of full length recombinant Lum.

The biphasic healing rate constants were summarized in Table 1. Our previous studies have demonstrated that during wound healing there is a suppression of cell proliferation in migrating epithelial cells [43], [44]. We hypothesized that the biphasic healing kinetics of HTCE cells in complete medium and in the presence of Lum may result from the promoted cell migration at the early phase of healing while the accelerated second phase of healing may be derived from the combined effects of cell proliferation and migration.

### Recombinant Lum promoted cell migration at the wound edge

**Figure 2:**
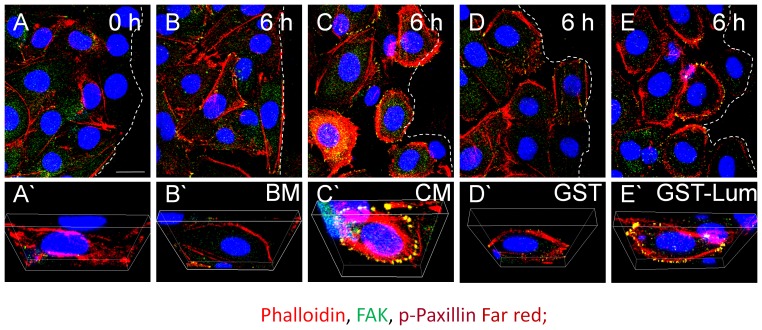
Lum promotes formation of adhesion complexes *in vitro.* Confluent HTCE cells were wounded and treated in various conditions for 6-D images of wounded HTCE cells (A–E) and 3-D images (A’-E’) showing expression/staining of F-actin, FAK and p-paxillin. In the presence of CM and GST-Lum, there is an increase in the number of adhesion complexes formed (yellow dots) when compared to treatment with BM and GST, consistent with promoted healing in HTCE cells treated with CM and GST-Lum (increased rate constants shown in Table 1). Dashed lines represent the wound edge. Phalloidin, red; FAK, green; p-paxillin, far red. Scale bar = 20 µm.

Lum promoted cell adhesion and locomotion is required for PMN extravasation during inflammation and corneal wound healing [33], [45]. Lum has also been shown to promote epithelial cell adhesion and migration [46]. Additionally, Lum has a role on tumor cell migration, through cytoskeletal rearrangement [47]–[49]. Therefore, attempts were made to find out if Lum may also have a role in epithelial cell migration during the healing of scratched HTCE cells *in vitro*. As shown in Fig. 2, six hours after a scratch wound there was an increase of adhesion complexes found in the presence of Lum and CM as determined by immunofluorescence staining of phospho-paxillin and FAK. In contrast, BM and administration of GST failed to promote the formation of such adhesion complexes. The role of lumican and C-terminal peptides of Lum on epithelium migration of debrided cornea is to be further examined *in vivo* using *Lum* ^−/−^ mice as described below.

### Recombinant Lum promoted wound healing was accompanied by sustained activation of pERK1/2 and lifted suppression of cell proliferation at the wound edge

**Figure 3:**
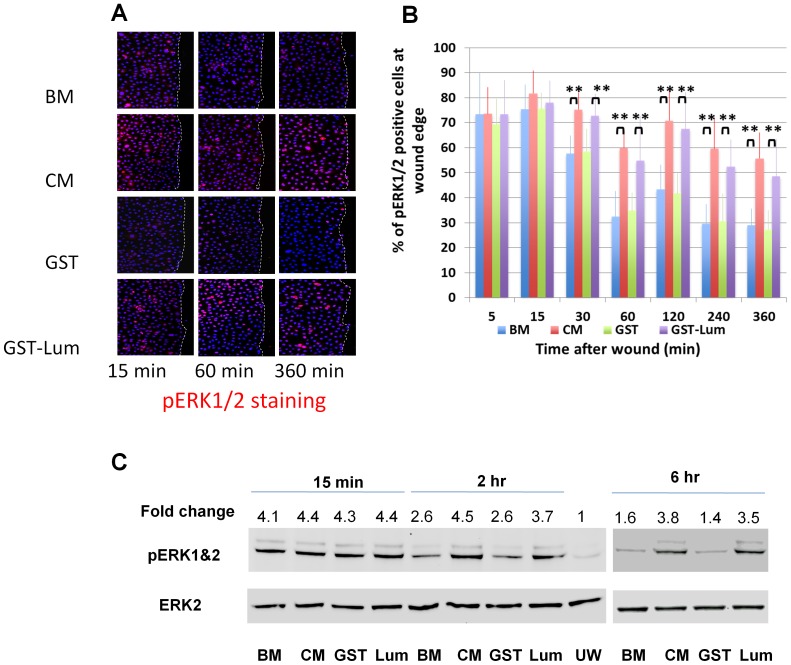
Lum sustained pERK1/2 activation at the wound edge of HTCE cells. HTCE cells were wounded and subjected to immunostaining with an anti-pERK1/2 antibody. (A) *Representative images of pERK1/2 immunostaining at different time points after wounding*. pERK1/2 was detected 15 min after wounding in all treatments. Expression decreased 2–6 h after wounding with BM and GST treatments. In contrast, expression was sustained out to 6 hours in the presence of GST-Lum or CM. (B) *Analysis of the percent of pERK1/2 positive cells at the wound edge.* In CM and GST-Lum, more pERK1/2 positive cells are present between 30 min and 360 min. (C) *Western blot analysis of pERK1/2.* In BM and BM+GST, there is a transient activation of pERK1/2 15 min after wounding. Activation of pERK1/2 decreased at 2 and 6 h after wounding. In contrast, the activation of pERK1/2 is sustained in the presence of CM and GST-Lum up-to 6 h after wounding. Densitometric measurements are compared to unwounded (UW) in basic medium. Statistical analysis was performed using ANOVA. Double asterisks represent p <0.01.

Previous reports indicate that overexpression of Lum in HTCE cells or pancreatic carcinoma activates Erk [46], [50]. Therefore, we attempted to examine if recombinant Lum could also stimulate the activation of ERK via phosphorylation during the healing of scratched HTCE cells. As shown in Fig. 3A, fifteen minutes after wounding, immunofluorescence staining showed that activation of pERK1/2 signal was observed in all treatments. The signal for pERK1/2 activation decreased 2–6 h after wounding in HTCE cells treated with BM and GST. In contrast activation of pERK1/2 was sustained for 6 hours in cells cultured in the presence of GST-Lum and CM. Statistical analyses are presented in Fig. 3B. Western blot analysis was performed to verify the observation. As shown in Fig. 3C, the sustained levels of pERK1/2 was seen in the presence of CM or GST-Lum for up to 6 hours, whereas the level of pERK1/2 initially increased at 15 min and then decreased 2–6 h after healing in cells treated with BM or GST.

**Figure 4:**
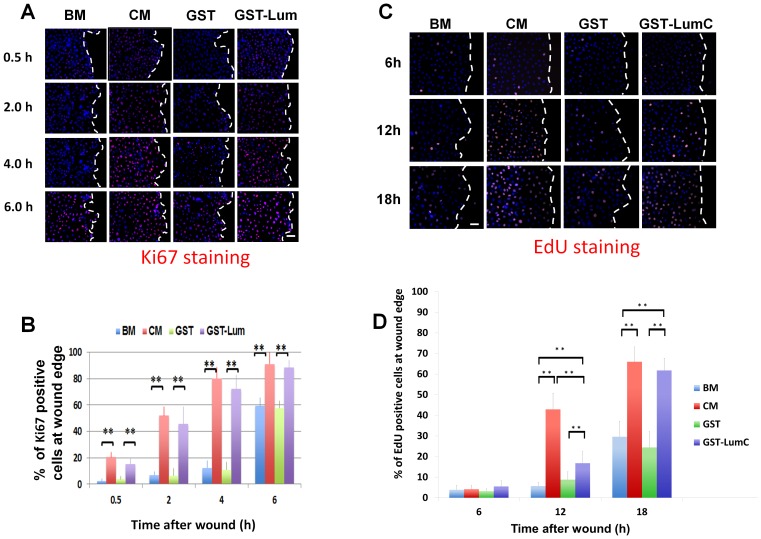
Administration of Lum lifts cell cycle suppression at the wound edge. Expression of Ki67, a marker of cells engaged in the cell cycle, was determined by immunostaining with an anti-Ki67 antibody at various time periods following wounding. (A) *Images of Ki67 expression pattern* Treatment with BM or BM+GST contained few Ki67 positive cells at the wound edge 0.5, 2 and 4 h after wounding but showed an increase in expression at 6 h. Treatment with CM and GST-Lum resulted in many Ki67 positive cells at all time points examined. Scale bar = 50 µm. (B) *Graphical representation of the percentage of Ki67 positive cells at the wound edge* (average±std, n = 12). (C) *Representative images of EdU-labeled cells (cells in S-phase) at the wound edge.* Few cells at the wound edge were EdU positive at 6 h and 12 h under the treatment of BM and BM+GST, while many EdU positive cells were seen in cells treated with CM and GST-LumC. Scale bar = 50 µm (D) *Graphical representation of the percentage of EdU positive cells at the wound edge* (mean±std, n = 4). No significant difference was seen at the 6 h time point. More cells at the wound edge enter S-phase 12 hours after wounding in CM and GST-Lum than those with BM and GST treatment. Statistical significance was analyzed by ANOVA. P value of<0.05 was considered significant and indicated by double asterisks. Dashed white lines mark the wound edge.

Lum purified from amniotic membrane is known to enhance cell proliferation accounting for the accelerated wound healing of corneal epithelium debridement *in vivo* and *ex vivo* [38]. Herein, we examined if recombinant Lum from *E. coli* also has that activity. A scratch wound assay was performed and expression of Ki67, a marker for cells in all phases of the active cell cycle, was determined. As expected, administration of GST-Lum and CM resulted in more Ki67 positive cells seen at the wound edge; in contrast BM or GST treatment contained few Ki67 positive cells (Fig. 4A and 4B). To determine if treatment of Lum does indeed result in enhanced cell proliferation, HTCE cells were subjected to a scratch wound in various culture conditions as described above. At different time intervals, the scratched HTCE cells were pulse labeled with EdU (5-ethynyl-2′-deoxyuridine) for 2 h prior to fixation with 4% paraformaldehyde as described in Methods. The number of EdU positive cells at the wound edge (100 µm from wound edge) were counted with an inverted microscope (ZEISS Axio Observer Z1 Apo Tome). As shown in Fig. 4C and 4D, in the presence of CM and GST-LumC_50_ recombinant protein the number of EdU positive cells increased in scratched HTCE cells 12 and 18 h after wounding. In contrast in the presence of BM and GST, a slight increase in the number of EdU positive cells was only seen at 18 h. These observations substantiate the hypothesis that the accelerated second phase of healing in scratched HTCE cells is a combined effect of cell migration and proliferation in the presence of CM and LumC.

### ALK5 is a Lum-binding protein

**Figure 5:**
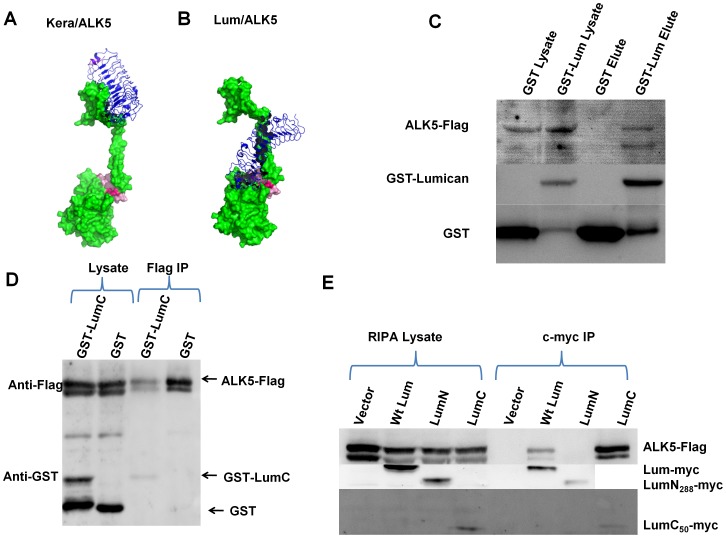
Lumican binds to ALK5: in silico analysis and pull down assays. In silico molecular dynamic analysis was performed to identify a binding partner of Lum. *Panels A and B represent potential binding of Kera and Lum to ALK5 (TGFβ type 1 receptor), respectively.* ALK5 in green and the GS domain of ALK5 (TGFbR1) in pink. (A) Keratocan interacts via its Leucine Rich Repeat domain with the extracellular domain of ALK5 with low affinity (ΔG = 30.39 kJ/mol). (B) Lum favorably interacts via its C-terminal domain with the GS domain of ALK5 (ΔG = −100 kJ/mol). The LumC domain has an even higher affinity with ALK5 (ΔG of −1086 kJ/mol). (C) *GST pull down assay shows in vitro interaction between ALK5 and GST-Lum.* ALK5-Flag was co-eluted with GST-Lum, but not GST, suggesting a direct interaction between Lum and ALK5. (D) *In vitro interaction between ALK5 and the C-terminal Lumican.* Both ALK5-Flag and GST-LumC were visible in lysates incubated with GST and GST-LumC. IP with an anti-flag antibody resulted in a GST positive band in the mixture of GST-LumC_50_ and cell lysate, but not in the mixture of GST and lysate, indicating that GST-LumC_50_, but not GST, is co-precipitated with ALK5-flag by anti-flag antibodies. (E) *C-terminal of Lum binds to ALK5.* ALK5-Flag was co-precipitated with LumC_50_-myc and Lum-myc, but not LumN-myc.

Results from our previous studies suggest that there is a cell surface receptor which mediates the matrikine functions of Lum [2]. Conventional affinity purification with cell extracts prepared from cultured cells incubated with immobilized Lum column failed to identify the perspective Lum receptor (our unpublished data). We and others have reported the role of Lum modulating TGFβ signaling, such as epithelial-to-mesenchymal transitions and Smad phosphorylation [37], [51]. We, therefore, performed molecular docking followed by 20 ns dynamic simulation in search of potential binding partners of Lum in the TGFβ pathway, such as TGFβs and TGFBR1 (ALK5). Fig. 5A and 5B show a model of the binding of Lum and Kera to ALK5 as predicted by molecular docking, suggesting that the C-terminus of Lum interacts with the GS-domain of ALK5 (ΔG = −100 kJ/mol), whereas the LRR domain of Kera weakly interacts with the extracellular domain of ALK5 (ΔG = 30.39 kJ/mol). It is of interest to note that the molecular dynamic data predicts a strong interaction between LumC_50_ and the GS domain of ALK5 with a ΔG of −1086 kJ/mol. This observation suggests that the C-terminus of Lum may mediate the binding to ALK5. The outcome of *in silico* analysis is consistent with the finding that GST-LumC_50_ promoted healing of scratched HTCE cells as shown in Fig. 1E and 1F, and Table 1. The data of molecular dynamics also indicate that Lum does not significantly bind to TGFβ1, 2 and 3 with ΔG of 82, 31 and 44 kj/mol, respectively. This observation also suggested that the Lum and LumC_50_ effects on HTCE wound healing was not due to the sequestration of TGFβs by Lum and LumC_50_.

The interaction between Lum and ALK5 was then biochemically verified through a series of reciprocal pull-down experiments. First, we employed a direct GST pull-down assay. HEK293 cells transfected with ALK5-Flag plasmid were incubated with GST-Lum at 4°C for 1 h and then treated with a reversible cross-linker Dithiobis Succinimidyl Propionate (DSP) to stabilize the potential protein complex of GST-Lum/ALK5-Flag or GST/ALK5-Flag. DSP has dual amine reactive groups covalently bonded by disulfide. Thus, it cross-links two closely interacting proteins through covalent bonds of the free amine of lysine residues in the proteins. DSP was used in all pull down experiments. The complex was subsequently isolated by a glutathione column and subjected to Western blotting in the presence of a reducing agent as described in the Methods. As shown in Fig. 5C, ALK5-Flag was co-eluted with GST-Lum, but not GST, suggesting a direct interaction between Lum and ALK5. GST-Lum was used to obtain enough recombinant Lum from *E. coli* for biological studies. To verify Lum/ALK5 interaction in HEK293 cells, a smaller epitope peptide (c-myc) was used to tag the Lum and its derivatives. Next, we investigated the ALK5-binding ability of different Lum domains. In this experiment, HEK293 cells were co-transfected with ALK5-Flag plasmid and c-myc tagged full length Lum (pSecTag-c-myc-Lum), N-terminal Lum (LumN) or C-terminal-Lum (LumC_50_) plasmids. The cell lysates were immunoprecipitated with anti-myc antibodies and subjected to Western blot with anti-Flag and anti-myc. ALK5-Flag was co-precipitated with LumC_50_-c-myc and Lum-c-myc, but not LumN-c-myc (Fig. 5E). As molecular docking predicted the C-terminus of Lum interacted with ALK5, we next used Flag immunoprecipitation to see if GST-LumC_50_ could be co-immunoprecipitated with ALK5-Flag. HEK293 cells were transfected with ALK5-Flag plasmid DNA. The cell lysate was incubated with GST-LumC_50_ and subjected to IP with an anti-Flag antibody immobilized bead. Fig. 5D shows that GST-LumC_50_, but not GST, is co-precipitated with ALK5-Flag. Caution should be taken when interpreting data from pull down experiments using cross-linking agents, because these agents have a tendency to increase non-specific interactions between proteins. The key to resolving this issue is to set up proper controls. In our experiments GST or c-myc-tagged LumN served as the control. Our data clearly show that ALK5 does not bind to GST or LumN, which supports our argument that the interaction between ALK5 and Lum is not an artifact caused by the cross-linker DSP. Together these data provide direct physical evidence showing that Lum does indeed bind to ALK5.

### Lum functions via binding to ALK5

**Figure 6:**
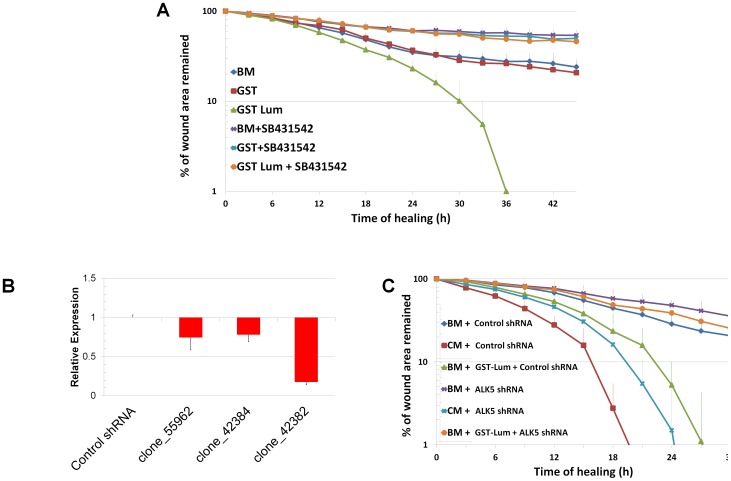
Inhibition and down regulation of ALK5 abrogates the effect of Lum on healing. (A) *ALK5-specific chemical inhibitor (SB431542) blocked the wound healing activity of Lum.* SB431542 completely blocked the wound-healing activity of GST-Lum. R^2^ values were as follows: BM 0.984; GST 0.986; GST-Lum 0.989; BM+SB431542 0.853; GST+ SB431542 0.958; Lum+ SB431542 0.920 (B) *Selection of ALK5 shRNAi clone.* A virus of scrambled control RNA did not change ALK5 mRNA level while ALK5-shRNAi down regulated the target mRNA level from 20% to 80%. The clone (42382) with an 80% knock-down efficiency was used in further experiments. (C) *Effects of shRNAi on wound healing of HTCE cells.* Down regulation of ALK5 by shRNAi did not significantly affect the biphasic healing of wounded HTCE cells in CM while it abolished the promotion of biphasic wound healing in GST-Lum. Control shRNAi did not affect Lum mediated wound healing. R^2^ values were as follows: Lum+scamble 0.968; Lum+ALK5shRNA 0.917.The rate constants are summarized in Table 2.

To verify the role of ALK5 in Lum-mediated wound healing, loss-of-function studies were employed. A wound healing assay was performed with HTCE cells treated with an ALK5 inhibitor (SB431542). As shown in Fig. 6A, SB431542 blocked the wound healing activity of Lum. Next, we knocked down ALK5 through lentivirus-based ALK5-shRNAi. The pGIPZ based lentivirus also carries a GFP gene for convenient titration of the virus. Three clones of ALK5 shRNAi were purchased from Openbiosystem (Fisher-Thermo Scientific, Pittsburgh, PA). The plasmids were packed into viruses and the viruses were transduced into HTCE cells. Because both pGIPZ-based ALK5 shRNAi lentiviruses and HTCE cells were generated by transducing retrovirus also carrying puromycin resistance gene in addition of human telomerase [52], ten times more puromycin was used to enrich positive transduced cells. After one week of puromycin selection, 100% of the cells were GFP positive (data not shown). The knockdown efficiency was determined by qRT-PCR. Among the three clones examined, clone_42382 has over 80% knockdown efficiency (Fig. 6B). This clone was then chosen for further studies to elucidate the role of ALK5 in mediating Lum effect on wound healing. Fig. 6C showed that down regulation of ALK5 by shRNAi abolished the Lum effect on wound healing, while it did not significantly affect the healing of scratched HTCE cells in CM. These experimental results can be explained by the fact that CM contains TGFα, a ligand of EGFR, which can also promote wound healing independent from TGFβ signaling pathways [53]. These data support the notion that ALK5 is critical for the wound healing activity of Lum. Table 2 summarizes the rate constants showing biphasic healing kinetics of HTCE cells and the abrogation and display of monophasic kinetics with inhibition of ALK5 with SB431542 and down regulation of ALK5 expression by shRNAi.

**Table 2:** Effects of ALK5 inhibitor and shRNA on Wound Healing of HTCE cells.

Culture condition	Rate constant 1 Δ%/h	Rate constant 2 Δ%/h
Basic medium (9)	3.37±0.71	N.A.
Basic medium + ALK5 inhibitor (3)	1.82±0.15 (P<0.01)^a^	N.A.
GST (9)	3.39±0.68(P = 0.95)^a^	N.A.
GST + ALK5 inhibitor (3)	1.83±0.17 (P<0.01)^a^	N.A.
GST-Lum (7)	5.37±1.17(P<0.01)^a^	14.83±1.61
GST-Lum+ALK5 inhibitor (3)	1.89±0.33 (P<0.01)^a^ P<0.01)^b^	N.A.
Complete medium (9)	6.12±0.85(P<0.01)^a^	16.86±1.71
Complete medium + control RNA (3)	5.72±0.86	17.28±0.45
Complete medium + ALK5 shRNAi (3)	5.09±0.83(P = 0.40)^c^	16.33±1.44(P = 0.34)^c^
Basic medium + control RNA (3)	3.45±0.63	N.A.
Basic medium + ALK5 shRNAi (3)	2.94±0.32 (P = 0.27)^d^	N.A.
GST-Lum + control RNA (3)	5.29±0.85	16.1±0.93
GST-Lum + ALK5 ShRNAi (3)	2.85±0.71 (P<0.05)^e^	N.A.

^a^ compared to basic medium; ^b, c^ compared to GST-Lum; ^d^ compared to complete medium + control RNA; ^e^ compared to basic medium + control RNA; ^f^ compared to GST-Lum + control RNA. P value <0.05 is considered significant.GST – Glutathione S-transferase. Statistical analysis using one way ANOVA:

### Lum C-terminal peptides stimulated wound healing both *in vitro* and *in vivo*

**Figure 7:**
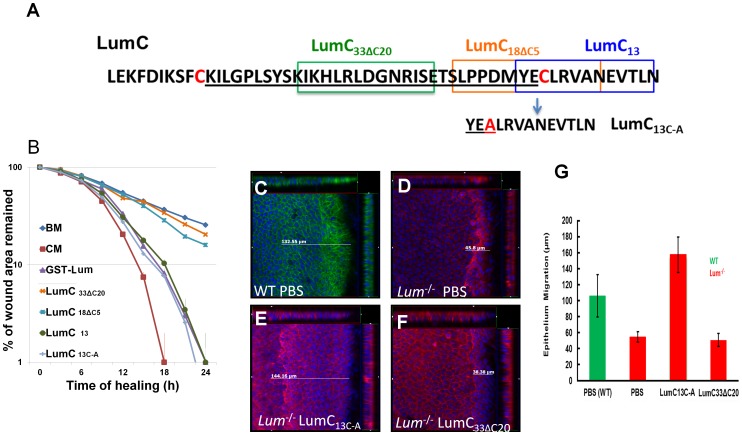
Identification of Lumikines (Lum C-terminal peptide(s) capable of stimulating wound healing in vitro and in vivo. (A) Amino acid sequences of synthetic LumC peptides, LumC_33_Δ_C20_, LumC_18_Δ_C5_, Lum_C13_ and LumC_13C-A_ (substitution of C with A). (B) In vitro analysis of the function of LumC peptides on the healing of HTCE cells. Healing followed biphasic kinetics in cells treated with CM, BM+GST-Lum, BM+Lum_C13_ and BM+LumC_13C-A_; whereas those of BM, BM+LumC_33_Δ_C20_ and BM+LumC_18_Δ_C5_ followed monophasic kinetics. The R^2^ values were as follows: full_Lum 0.993; LumC_18ΔC5_ 0.970; LumC_33ΔC20_ 0.936; LumC_13_ 0.952; LumC_13c-a_ 0.902. These data suggest that the last C-terminal 13 amino acids have wound healing activity. (C–G) In vivo wound healing activity of Lumican peptides. To confirm LumC_13C-A_ can also promote corneal epithelium wound healing in vivo, Lum^-/-^ mice were subjected to epithelium debridement. C–F are representative cut view images showing the migration distance. Migration distance was measured between the original wound edge and the wound front. In LumC_13C-A_ treated corneas, the wound front moved ∼144 µm. The control peptide, LumC_33_Δ_C20_, did not promote epithelium wound healing. (G) Summary of epithelium migration. The average epithelium migration (µm) and standard deviation were calculated from five corneas of two separate experiments. In PBS and peptide LumC_33_Δ_C20_ peptide treated cornea, the epithelium moved ∼38 µm, while corneas treated with LumC_13C-A_ migrated ∼140 µm. Injured corneal epithelium of wild type mice migrated about 100 µm.

Molecular docking predicted that the C-terminal domain of Lum binds to ALK5 (Fig. 5B) and biochemical pull down assays also verified the role of LumC_50_ in the ALK5 interaction (Fig. 5C, 5D and 5E), in addition GST-LumC_50_ was capable of promoting HTCE wound healing (Fig. 1 and Table 1). However, the minimal domain of Lum essential for promoting wound healing remains elusive. Wound healing assays were then performed using chemically synthesized peptides covering different regions of the C-terminus of Lum. LumC_13_ (the last 13 amino acids at the C-terminus of Lum) is capable of promoting wound healing of scratched HTCE cells (Fig. 7A). LumC_13_ contains a cysteine residue, which renders it susceptible to oxidation and dimer formation via bisulfite-bond, resulting in loss of its activity. Our mass spectrometry data verified the existence of such a LumC_13_ dimer in solution (data not shown); therefore, the cysteine residue was substituted with alanine. The resulting peptide (LumC_13C-A_) is effective in promoting wound healing with similar efficacy as full length Lum and LumC_50_. No significant difference in the rate constants was noted among Lum, LumC_50_, LumC_13_ and LumC_13C-A_ (Table 1). Interestingly, both LumC_18_Δ_C5_ and LumC_33_Δ_C20_ that lack the last 5 C-terminal amino acids EVTLN do not promote the healing of scratched HTCE cells (Fig. 7A and Table 1).

We have previously demonstrated that the addition of Lum can promote the healing of epithelium debridement in *Lum* ^−/−^ mice by enhanced cell proliferation and reduction of apoptosis [3]. It is not known, however, whether C-terminal peptides of Lum can promote epithelium migration *in vivo*. To examine this possibility, epithelium debridement in *Lum* ^−/−^ mice were treated with eye drops containing LumC peptides. Epithelial debridement wounds were made in adult *Lum* ^−/−^ mice, after which, PBS, LumC_33_Δ_C20_ and LumC_13C-A_ were applied to the eyes *via* eye drops every 15 min for 2 h. Wild type mice were used as controls. Corneas were collected and whole mount staining was carried out with phalloidin and DAPI. There is a morphological change in the injured cornea; the removal of epithelium causes a slight elevation of stroma at the denuded corneal surface possibly due to stromal hydration. For example, Fig. 7C shows a cut view of Z-stacked images of debrided cornea from a wild type mouse which healed for 2 h. The epithelium migration was greatly retarded in the healing epithelium of *Lum* ^−/−^ mouse (Fig. 7D). Treatment with LumC_13C-A_ greatly improved the epithelium migration (Fig. 7E), whereas administration of LumC_33_Δ_C20_ had little effect on wound healing (Fig. 7F). Fig. 7G summarized the data from four different treatments with each treatment including a total of five eyes from two separate experiments. Administration of LumC_13C-A_ also promoted wound healing of epithelium debridement in wild type mice to the same extent of injured corneas of *Lum* ^−/−^ mice treated with LumC_13C-A_. These results showed that the LumC_13C-A_ peptide significantly promoted epithelium wound healing in both wild type and Lum^−/−^ mice.

## Discussion

### Lum promotes wound healing via interaction with ALK5

In this study, we have demonstrated that recombinant GST-Lum and GST-LumC_50_ fusion proteins purified from *E. coli*, which lack all eukaryote-specific post-translational modifications, promote biphasic wound healing kinetics of scratched HTCE cells *in vitro*. Furthermore synthetic lumikines (Lum derivative peptides regulating cell activity), e.g., LumC_13_ and its LumC_13C-A_ derivative are capable of promoting similar wound healing both *in vitro* and *in vivo*. We have shown that this promotion of wound healing is attributed to the interaction of Lum to ALK5.

### Cell migration and proliferation

During the early phase of corneal epithelium wound healing, cell proliferation is suppressed at the migrating edge of the epithelium [43], [44], [54]. In the present study, we observed sustained Erk phosphorylation at the wound edge that was accompanied by lifted cell cycle suppression as seen from Ki67 expression in GST-Lum treated HTCE cells as shown by western blot and immunofluorescence staining. To determine the number of cells in the S-phase, EdU incorporation was examined and showed that there was an increase in the number of EdU-labeled cells at the wound edge of scratched HTCE cells at 12 h of healing in the presence of Lum and CM. In contrast, HTCE cells treated with BM did not have an increase in the number of EdU-labeled cells until 18 h of healing. The increase of cells in S-phase in CM was slightly increased to that of Lum-treated HTCE cells, an observation consistent with the notion that cell proliferation at the wound edge had a significant effect on wound healing.

### TGFβ signaling during wound healing

The results of *in silico* analysis, pull down assays and HTCE wound healing in the presence of ALK5 inhibitor SB431542 and ALK5-shRNAi suggest that the Lum/ALK5 complex plays a significant role on epithelium wound healing albeit the underlying molecular mechanism is still not known. In response to a wound, cells release TGF-β to facilitate the healing process [55]–[57]. During epithelium wound healing, several TGF-β pathways can potentially be activated; these include Smad, p38MAPK and ERK [44], [58]–[60]. Cell cycle arrest and cell migration are two important events during the initial phase of wound healing. Cell cycle arrest can be mediated by the Smad-dependent pathway and phosphorylation of ATF2 by p38MAPK and JNK pathways [44], [60], [61]. In the absence of ligand binding, ALK5 binds to inhibitory immunophilin FKBP12 in the GS region to block ligand-independent phosphorylation of ALK5 by TGFBR2 [62], [63]. Upon TGF-β stimulation, ALK5 is phosphorylated at the GS region by TGFBR2. FKBP12 is replaced by Smad2 which is subsequently phosphorylated by ALK5 [64]. Phosphorylated Smad2 then polymerizes with Smad 4, the Smad2/Smad4 complex is transported into the nucleus to drive the expression of the cell cycle arrest gene p15. Meanwhile, the Smad-independent Erk pathway can also be activated by TGF-β. TGF-β induces phosphorylation of tyrosine residues on both ALK5 and ShcA. The domain containing phosphorylated tyrosine is then capable of recruiting Grb2/Sos to activate Erk through Ras, Raf, and their downstream MAPK cascade. Erk then regulates target gene transcription through its downstream transcription factors to promote cell migration [65]. Further studies are needed to examine this possibility. It also waits to be proven if Lum/ALK5 effects on epithelium wound healing may be mediated through pathways independent of TGFBR2. For example, Lum/ALK5 may trigger a signal transduction cascade similar to what has been described for the direct ligand binding of ALKs as is seen in the case of IL-13 inducing CTGF expression in HSCs by activating TGF-β–independent activin receptor-like kinase/Smad signaling via the Erk-MAPK pathway [66]–[68].

Currently, we do not know the exact mechanism by which Lum promotes wound healing through ALK5. There are at least two possibilities that may account for the Lum function on epithelium wound healing and can be examined in future studies: 1). The binding of Lum and its C-terminal peptides to the GS-domain of ALK5 may interfere with the phosphorylation of Smad by ALK5 after TGF-β binding to TGFBR2; 2). The Lum/ALK5 complex triggers signaling cascades that are independent of canonical signaling cascades of TGFBR2 binding. Furthermore, there is a remote possibility that the binding of Lum to ALK5 may serve as a mechanism to attenuate TGF-β signaling pathways via the internalization of the Lum/ALK5 complex similar to what has been reported for decorin binding to EGFR [69], [70]. Further studies are needed to examine these possibilities.

In summary, we found and verified the long-sought receptor of Lum. Several observations made in this study also lead us to a tentative model as described above, which can explain the phenomena reported in this and previous studies. Verification of the model will provide more insight into mechanisms not only on Lum function but also on TGF-β signaling as well. We also found that peptides from the C-terminal domain of Lum can promote wound healing both *in vitro* and *in vivo*, making them promising therapeutic reagents for epithelium wound healing.

## Materials and Methods

### Cell culture

HEK293 cells were purchased from Invitrogen (Carlsbad, CA) and maintained in DMEM (Gibco-Invitrogen) supplemented with 10% fetal bovine serum. HTCE Cells were originally prepared from human corneal epithelial cells immortalized by human telomerase. Characterization of the immortalized cell line was published in by Robertson, et al. (Invest Ophthalmol Vis Sci 46: 470–478, 2005) [52]. Primary culture epithelial cells were harvested individually from 21 donor eyes (ages range, 26–67 years), over a period of 5 months. All donor corneas were obtained from the Tissue Transplant Services Lions' Eye Bank (Dallas, TX) in accordance with the provisions of the Declaration of Helsinki for research involving human tissue. HTCE cells were maintained in serum free keratinocyte culture medium, Dermalife®K complete medium kit (Lifeline Cell Technology, Frederick, MD), supplemented with 6 mM L-Glutamine, 0.4% bovine pituitary extra, 1.0 µM Epinephrine, 0.5 ng/ml rh (recombinant human) TGFα, 100 ng/ml Hydrocortisone Hemisuccinate, 5 µg/ml rh Insulin and 5 µg/ml Apo-Transferrin. Medium was changed every 2 days.

### Preparation of GST-Lum, and GST-LumC fusion proteins

PCR was used to generate the mouse lumican full-length cDNA digested with Mfe1 (compactable to E.CoR1), which was cloned into the EcoR1 site of pGEX-2T plasmid (GE Healthcare, Piscataway, NJ). The sequences of PCR primers are as following:

LumF GGCCAATTGCCATGAATGTATGTGCGTTCTCTCTTG;

LumR GCCGCAATTGTTAGTTAACGGTGATTTCATTTGC

GST-LumC was generated by cloning PCR product into BamHI/EcoRI site of pGEX-2T using the following primer set:

Lum1262F GAGGAAGCTTCTTGAAAAGTTTGATGTGAAG and LumR. The origami (DE3) *E. coli* strain (Novagen-EMD Chemicals, Philadelphia, PA) was transformed with the plasmids. Recombinant bacteria were grown in Luria-Bertani medium containing ampicillin (50 µg/ml), kanamycin (15 µg/ml) and tetracycline (12.5 µg/ml) at 37°C to a density of 0.6–0.8 A600. IPTG was then added to a final concentration of 0.05 mM and protein expression was allowed to proceed for 4 h at room temperature. The bacteria were then collected by centrifugation (5,000 rpm, 10 min) and lysed for 30 min in STE buffer (10 mM Tris-HCl, 1 mM EDTA, 150 mM NaCl, pH 8.0) with lysozyme (100 µg/ml). To the specimens added 1 mM DTT, 0.1 mM PMSF, cocktail of protease inhibitors (x100) and 0.5% N-Lauroylsarcosine, the lysates were sonicated, power 3.5, total 1.5 min, and then centrifuged for 20 min at 16,000 rpm. Triton-X100 was added to the supernatants to 0.5% and the mixtures were loaded on glutathione Sepharose columns. After elution with 20 mM glutathione in 50 mM Tris-HCl, pH 8.0, purified GST fusion protein was checked by Coomassie Brilliant Blue staining and western blot. Purified protein was dialyzed with PBS overnight and stored in −80°C.

### pSecTag-Lum-c-myc, LumC_50_-c-myc and LumN_288_-c-myc plasmids

Mammalian expression vector expressing full length lumican (pSecTag-Lum-c-myc) was constructed as reported previously [71]. N-terminal deletion (LumC50-c-myc) and C-terminal deletion (LumN288-c-myc) were made by HindIII digestion together with SfiI or NotI, respectively.

### Molecular modeling and docking of Lum and molecular dynamics

A 3D-model of the lumican and keratocan molecules were built using MODELLER 9v1, based on the structure of DCN (PDB 1XCD) as a template [72], [73]. The best structure for each molecule was selected using the Discrete Optimized Protein Energy (DOPE) model score [74], GROMACS simulation suite, version 4.5.1 [75] and GROMOS96 43a1 force field [76], [77].

Docking calculations of the modeled Lum and Kera, intracellular ALK5 kinase domain (pdb ID 2X7O)[78], extracellular domain (pdb ID 3KFD) [79], decorin (pdb ID 1XEC), TGFβI (pdb ID 3KFD), TGFβII (pdb ID 2PJY) and TGFβIII (pdb ID 1TGJ) were performed using Hex 4.5 the Macro Docking option [80]. The HEX docking algorithm employs molecular shape comparison using spherical harmonics and polar Fourier correlations. The docking calculation was performed using 57 intermolecular separations in steps of 0.075 nm. A total of 200 docking solutions were obtained and post-processed using the bump option to calculate the number of steric clashes in each solution. The low resolution spherical harmonic representation of the receptor was kept at 5. The top five ranked clustered solutions were analyzed and the lowest energy complex was submitted to molecular minimization (http://hex.loria.fr/). This provides visualized simulation of docked complexes.

Molecular dynamics (MD) simulation, trajectory and interaction energy analysis were performed using the GROMACS simulation suite employing united atoms gromos96 43a1 force field, as described elsewhere [81]. The docked complexes were solvated in rectangular boxes using periodic boundary conditions and SPC water model [82]. Counter ions (Na+, Cl^−^) were added to neutralize the system, whenever needed. The employed MD protocol was based on previous studies [83]. The Lincs method was applied to constrain covalent bond lengths [75], [84], allowing an integration step of 2 femtosecond (fs) after an initial energy minimization using Steepest Descents algorithm. Electrostatic interactions were calculated using Particle Mesh Ewald method [85]. Temperature and pressure were kept constant by coupling protein complexes, ions and solvent to external temperature and pressure baths with coupling constants of τ = 0.1 and 0.5 ps [86], respectively. The dielectric constant was treated as ε = 1. The systems were heated slowly from 50° to 310°K, in steps of 5 ps, each one increasing the reference temperature by 50°K. After this heating, all simulations were further extended to 20 ns under a constant temperature of 310°K. Hydrogen bonds were defined when the donor-acceptor heavy atom distance was 0.35 nm and the acceptor atom–donor hydrogen angle was 30 degrees. The final structures of the complexes were analyzed with Pymol and VMD [87].

### Preparation of stable shRNAi-ALK5-HTCE transformants

HTCE were infected with scrambled control shRNA or ALK5-shRNAi lentivirus (pGIPZ clones V2LHS_42382, V2LHS_55962, V2LHS_42384), which were packaged with Trans-Lentiviral™ Packaging Kit (Openbiosystems, Thermo Fisher Scientific, Inc). Stable ALK5-shRNAi-HTCE transformants were selected with 20 µg/ml puromycin. ALK5 expression was verified using qRT-PCR.

### Scratch wound healing of HTCE cells

HTCE cells and ALK5-shRNAi transformants were inoculated in 24 well culture plates and grown to confluence in complete medium. Confluent cultures of the cells were starved in basic medium supplemented with 6 mM L-glutamine for 24 h and then subjected to a scratch wound in the presence of either recombinant GST-Lum (10 µg/ml = 0.15 µM) and GST-LumC_50_ (C-terminal regions of Lum, at 0.15 µM), GST (10 µg/ml) recombinant proteins in basic medium. Complete medium was used as a positive control and basic medium as a negative control. The wound healing rate of the scratch wound was determined by calculating the mean area of the cell-gap which was imaged every 3 h until wound closure; therefore, 100% at 0 h and 0% at complete wound closure, using a Time Lapse microscope (Axio Observer A1, ZEISS). The cell-gap area was calculated using Axio vision software (Zeiss, Vertrieb, Germany). Three images were collected for each experimental point and the experiment was carried out in triplicates. The percentage of cell-gap area at each time point was normalized by the cell-free area at 0 h, and the values were plotted on a semi-log chart as a function of time. The rate of healing is calculated as “healing rate constant = Δ%(%_t+6_ - %_t)_ ÷ 6 h) = Δ%/h) ” where Δ is the percentage difference of cell gap between any two time points 6 h apart and t is any time point within the first phase or second phase. The method for determining the “goodness of fit” for this kinetic model is given in the Statistical Analysis section below.

Confluent cultures of HTCE were starved in basic medium supplemented with 6 mM L-glutamine for 24 h. Then cells were pretreated with SB431542 (10 µM) (Reagentdirect, Encinitas, CA) overnight and scratch wound was performed in the presence of either GST-Lum (10 µg/ml) or GST (10 µg/ml). Complete and basic medium were used as controls.

### Lum effects on healing of epithelium debridement *in vivo*

Animal care and use conformed to the ARVO Statement for the Use of Animals in Ophthalmic and Vision Research. All animal protocols were approved by the Institutional Animal Care and Use Committee (IACUC) of the University of Cincinnati. Lum knockout mice (*Lum* ^−/−^) (8 weeks old) were anesthetized by intraperitoneal injection of ketamine hydrochloride (5 µg/gm body weight) and xylazine (0.625 µg/gm body weight). Epithelium debridement was made with Alger-Brush®. PBS, LumC_33_Δ_C20_ and LumC_13C–A_ were applied as eye drop every 10 min for 2 h. Corneas were collected and fixed in 4% paraformaldehyde (PFA) in PBS at room temperature for 30 min. Excess PFA was removed by wash and quenched with 0.1% NaBH_4_, and whole mount stained with phalloidin and DAPI at 4°C overnight. Images were taken with a Zeiss Apo Tome microscope (Observer Z1). The removal of epithelium leads to a slight elevation of stroma at the wound edge due to hydration. The front moving distance can be measured between the moving front and original wound edge.

### Immunofluorescence

Confluent HTCE cells were cultured in 2-well chamber slides (Thermo Fisher Scientific Inc. Pittsburgh, PA). After a 24 h starvation in basic medium, a linear wound was produced in the monolayer with a 1 ml pipet tip. The cells were allowed to heal for different period of times as indicated in Figures with basic medium (BM), complete medium (CM), GST-LumC_50_ (0.15 µM) or GST (10 µg/ml) in the basic medium. The cultured cells were fixed with 4% paraformaldehyde in PBS for 30 min and then subjected to immunofluorescence staining with either anti-Paxillin (Cell Signaling, Danvers, MA), Ki67 (Abcam, Cambridge, MA) and pERK1/2 (Cell Signaling) antibodies according the manufacturers' instructions, counter stained with phalloidin, and examined with a fluorescence microscope (Axio Observer Z1, ZEISS) as previously described [44].

### Western blotting of pERK1/2

Confluent HTCE cells were incubated in basic medium for 24 h, 8 linear scratches intersecting at the center of the culture dish were produced with a 1 ml pipet tip. The cells were allowed to heal for various periods of times in the presence of GST-Lum (10 µg/ml) or GST (10 µg/ml) in basic medium. Thereafter, cells were homogenized in lysis buffer (100 µl of CelLytic-M mammalian cell lysis/extraction reagent; Sigma) supplemented with protease inhibitors cocktail (Sigma). The cell lysate was clarified by centrifugation, mixed with 5× sample buffer, and subjected to SDS- PAGE in 10% acrylamide (sodium dodecyl sulfate-polyacrylamide gel electrophoresis), transferred to polyvinylidene difluoride (PVDF) membrane, and immunostained with anti-pERK1/2 and total ERK2 (Cell Signaling) as described previously[88].

### Pull-down assays

HEK293 cells were transfected with pRK5-ALK5-flag plasmid (plasmid 14831) (Addgene, Cambridge, MA) for 48 hours, cells were harvested and lysed in lysis buffer (0.3% CHAPS in PBS with Ca/Mg). Recombinant GST or GST-LumC_50_ was added to the lysate and incubated at 4°C for 1 h in the presence of 0.5 mg/ml Dithiobis succinimidyl propionate (DSP), a reversible crosslinking reagent (Thermo Fisher Scientific, Inc). Flag-tagged proteins were pulled down by anti-flag Sepharose-beads (Sigma) in lysis buffer. The beads were washed with lysis buffer 5 times and the protein bound was eluted with flag peptide. Samples were subjected to SDS-PAGE (10–20% acrylamide gradient) and were visualized by anti-flag and anti-GST antibody.

HEK293 were transfected with pRK5-ALK5-flag plasmid and cultured for 48 h. The transfected cells were cooled to 4°C and then incubated with GST-Lum (10 µg/ml) or GST (10 µg/ml) at 4°C for 1 h, and then incubated with 0.5 mg/ml DSP for 1 h. The cells were lysed in lysis buffer and the lysates were applied to glutathione-Sepharose column and washed with PBS, the bound proteins were eluted with glutathione (20 mM in PBS) and eluents were subjected to Western blot analysis in the presence of 1% MeSH as described above to dissociate the cross-linked proteins.

Alternatively, HEK293 cells were co-transfected with pRK5-ALK5-flag and pSecTag-Lum-myc, or pSecTag-LumN288-myc, or pSecTag-LumC_50_-myc plasmids, and cultured for another 48 h, the cells were treated with 0.5 mg/ml DSP on ice for 45 min and the reaction was stopped by adding 100 mM TrisHCl pH 7.4. The cell lysates were prepared with lysis buffer (50 mM Tris/HCl pH 7.4, 150 mM NaCl, 1% TritonX100, Protease Inhibitor Cocktail). Protein complex was pulled down by anti-c-myc Sepharose-beads (Sigma). The beads were washed with lysis buffer 5 times and then the bound ALK5-flag/LumC_50_-myc was eluted with c-myc peptide. Samples were subjected to SDS-PAGE in the presence of 1% β-mercaptoethanol and were visualized by anti-flag and anti-c-myc antibodies (Cell Signaling).

### EdU labeling

Cell proliferation was determined by EdU incorporation. HTCE cells cultured at different conditions were incubated with 10 µM of EDU in culture medium at 37°C for 2 h. EDU Cells were then fixed with 2% PFA and followed by adding cocktail (100 mM CuSO4, 1 M Sodium Ascorbate and 2 mM Azide) for 30 minutes at room temperature protected from light. Cells were also counterstained with DAPI. Fluorescence images were visualizing using fluorescence microscope as described above.

### Statistical Analysis

To determine the “goodness of fit” for the kinetics model we performed the following to obtain the R^2^ value. 1) Calculated the total sum of square (TSS): sum of the square of the distances of the points from a horizontal line through the mean of all Y values. 2) Calculated the sum of the squares of the distances of the points from the curve (SSC). 3) Used the following equation: R^2^ = 1-SSC/TSS [89].

All statistical analyses were performed using the StatPlus:macLE software for MacOSX (Analyst Soft, Alexandria, VA, USA). Data were statistically analyzed by ANOVA with Bonferroni post hoc analysis and P values of <0.05 were considered significant.
